# Feasibility, Acceptability, and Potential Efficacy of a Mobile Health Application for Community-Dwelling Older Adults with Frailty and Pre-Frailty: A Pilot Study

**DOI:** 10.3390/nu16081181

**Published:** 2024-04-16

**Authors:** Takahisa Ohta, Yosuke Osuka, Takashi Shida, Kaori Daimaru, Narumi Kojima, Kazushi Maruo, Ai Iizuka, Moe Kitago, Yoshinori Fujiwara, Hiroyuki Sasai

**Affiliations:** 1 Research Team for Promoting Independence and Mental Health, Tokyo Metropolitan Institute for Geriatrics and Gerontology, Itabashi 173-0015, Japansasai@tmig.or.jp (H.S.); 2 Department of Frailty Research, Center for Gerontology and Social Science, Research Institute, National Center for Geriatrics and Gerontology, Obu 474-8511, Japan; osuka@ncgg.go.jp; 3 Institute of Medicine, University of Tsukuba, Tsukuba 305-8575, Japan; 4 Research Team for Social Participation and Healthy Aging, Tokyo Metropolitan Institute for Geriatrics and Gerontology, Itabashi 173-0015, Japan

**Keywords:** mHealth, frailty, pre-frailty, feasibility

## Abstract

Smartphone applications aimed at enhancing physical, cognitive, and social activities through mobile health (mHealth) technology are of increasing interest. Their feasibility and acceptability, alongside impacts on frailty phenotype scores and step counts among older adults with frailty, remain to be fully validated. This study presents a 13-week preliminary intervention trial assessing an mHealth app’s feasibility in a cohort of 34 eligible older adults, including 5 frail and 29 pre-frail participants. The intervention entailed a 6-week course on app usage, followed by 7 weeks of observation, with four participants withdrawing early. Feasibility was determined by login and active use rates, with a target login rate of 60% or higher. Post-intervention, 100% session attendance and a median login rate of 88.4% were observed. Acceptability was high, with 73% affirming the app’s health benefits. Notably, frailty scores and step counts improved post-intervention, underscoring the app’s potential for supporting older adults with frailty.

## 1. Introduction

Frailty is characterized by exacerbated susceptibility, manifesting as an inadequate response to stressors, owing to a progressive decline in physiological stability across organ systems [1]. The theoretical framework of Fried et al. associates frailty with an increased risk of impaired mobility, frequent falls, functional limitations, hospital admissions, and mortality [2]. A reduction in physical activity, such as that seen especially among isolated older adults during the COVID-19 pandemic, exacerbates frailty [3]. Amid the debate on the operational definition of frailty assessment, there is a clear need for comprehensive and cost-effective intervention studies [4,5].

The Ministry of Health, Labor, and Welfare in Japan has promoted the establishment centers called “kayoinoba” (i.e., community gathering place) for essential older-adult community engagement in frailty prevention. By 2019, over 110,000 such communities were operational nationwide, bolstering community-based care and efforts to prevent frailty [6]. Approximately 18 million Japanese individuals aged 65 and older have been identified as pre-frail or frail, highlighting the urgent need for effective “kayoinoba” management strategies. However, limitations in human resources necessitate the development of sustainable approaches [7].

The increasing use of smartphones among older adults, coupled with advancements in digital technology—particularly mobile health (mHealth)—has spurred interest in digital tools for frailty mitigation [8]. The National Center for Geriatrics and Gerontology launched an “Online Kayoinoba” app aimed at increasing the activity levels of older adults and preventing frailty [9]. This app incorporates features designed to encourage and support walking, exercise videos, cognitive games, and health monitoring, effectively replicating the “kayoinoba” experience for home-based, self-managed activities.

Despite the initiation of large-scale randomized controlled trials (RCTs), such as the dementia prevention program by Shimada et al. [9], the impact of the “Online Kayoinoba” on health outcomes among older adults with pre-frailty or frailty remains to be fully understood. Significantly, few RCTs have targeted older adults with pre-frailty or frailty using mHealth apps, indicating a notable research gap [10]. To bridge this gap, large-scale clinical trials are essential to definitively ascertain the effectiveness of these apps; as a preliminary step, the feasibility and potential benefits of “Online Kayoinoba” deserve thorough exploration.

Therefore, this study aims to evaluate the feasibility, acceptability, and potential efficacy of the “Online Kayoinoba” app for community-dwelling older adults with frailty, intending to guide future RCTs toward broader implementation.

## 2. Materials and Methods

### 2.1. Design and Participants

This single-arm study unfolded over 13 weeks. Participants were selected from residents of Itabashi Ward, Tokyo, who had undergone a comprehensive medical examination by the Tokyo Metropolitan Institute for Geriatrics and Gerontology in October 2022. The inclusion criteria for participants were as follows: they had to be 65 years or older, identified as frail or pre-frail according to the revised Japanese version of the Cardiovascular Health Study (J-CHS) criteria [11], owners of a smartphone, and capable of providing informed consent. Exclusion criteria included individuals diagnosed with dementia, those undergoing treatment for dementia, those with contraindications to exercise, participants engaged in other mHealth app-based studies at our institute, and those unable to perform motor function tests or other assessments. No financial incentives were offered for participation in this study. The Human Research Ethics Committee of the Tokyo Metropolitan Institute for Geriatrics and Gerontology [R22-079] approved this study.

### 2.2. Intervention Program

**Figure 1:**
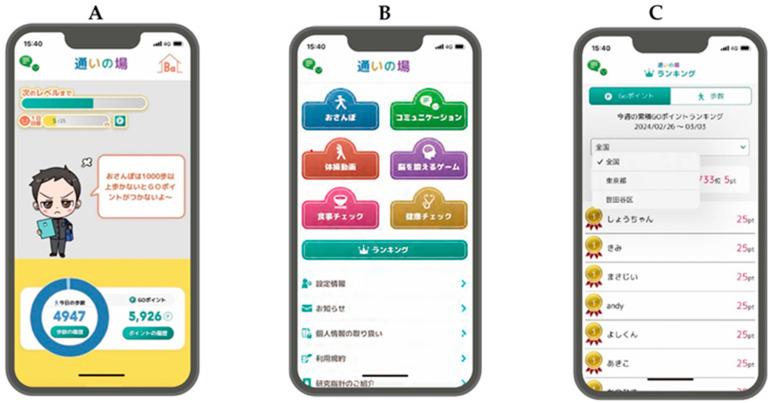
Screenshots of the mHealth application “Online Kayoinoba”. (**A**) Home Screen: This interface allows users to review and develop characters, as well as examine their messages. It also enables the tracking of daily step counts and the history of points earned. (**B**) Menu Screen: This screen provides access to six onboard functions. The Walking function, indicated by a blue tab, facilitates the generation of outdoor walking courses and the setting of goals for points, duration, and step counts. The Home Exercise function, shown with an orange tab, delivers community exercise videos specific to each municipality. Diet Control, represented by a pink tab, allows users to log meals and monitor meal balance. The Communication function, marked by a green tab, offers a chat feature for effortless communication among users and groups. Cognitive Training, identified by a purple tab, employs puzzles, calculations, and other gaming challenges to improve cognitive functions. Health Check-Up, displayed with a yellow tab, leverages the Home Activity Guide 2020 and the Kihon Checklist, enabling users to explore guidelines for exercises and activities suitable for home implementation, based on physical and cognitive abilities. (**C**) Ranking Screen: This display permits users to view their earned points and step counts within the rankings (at the national, prefectural, and municipal levels).

The features of the mHealth application, “Online Kayoinoba” (Figure 1), have been detailed elsewhere [9]. The “Online Kayoinoba” application is a preventive tool for long-term care designed to enhance physical activity duration and social interaction opportunities for seniors during COVID-19. It facilitates engagement in exercise, cognitive, and health promotion activities through online platforms. This application is available at no cost for both installation and use and can be downloaded from the Apple App Store or Google Play Store. Participants attended lectures at one time weekly, with each session lasting 60 min, resulting in an intervention period of six weeks. The content of the lectures, outlined in Table 1, aimed to familiarize participants with one function per lecture. All lectures were facilitated by researchers who regularly use the application and were structured to include a maximum of ten participants for every five staff members. In addition to the lectures, participants were provided with summaries of the covered content as part of the lecture materials for review at home. The six-week intervention phase was succeeded by a seven-week observation period, during which participants were encouraged to actively use the app without any direct communication with the researchers.

### 2.3. Sample Size

This feasibility study was not structured to assess significant differences, hence there were no formal criteria for sample size [12]. The objective was to enlist 30–40 participants.

### 2.4. Feasibility of mHealth Apps

The “Online Kayoinoba” application incorporates a point system known as “GO Points”, which users can accumulate by engaging with the app. Users are awarded one point for logging in and additional points for utilizing various features. This system encourages users to gradually increase their points by consistently logging in and engaging with different functions. The total number of “GO Points” accumulated can be monitored on each user’s screen.

The feasibility of the app was assessed by determining the percentage of days on which the app was logged into (with at least one point earned) and actively used (with at least seven points earned) throughout the entire intervention period (six weeks) and the subsequent observation period (seven weeks). Given that no prior studies have investigated the feasibility of mHealth apps for older adults with frailty, and there are no established criteria for measuring adherence, this study operationally defined feasibility as a login rate of 60% or higher. This was calculated by dividing the number of days logged in by the number of intervention days, drawing on evidence that web- and mobile-based remote exercise guidance typically achieved 67–84% adherence [13,14,15]. Other studies focusing on physical activity promotion through mHealth apps reported an adherence rate of 44.8% [16].

### 2.5. Acceptability

The acceptability of the app was evaluated by asking all participants to complete a self-administered questionnaire after their intervention, which included the following questions: (1) “Does the app help you improve your health?” and (2) “Would you recommend the app to your friends and family?”. Responses were measured on a five-point scale: “agree”, “somewhat agree”, “neither agree nor disagree”, “somewhat disagree”, and “not at all agree”. Responses of “agree” or “somewhat agree” were considered positive. Although a scale for evaluating the acceptability of mHealth apps exists, a Japanese version has not yet been developed, making it unavailable for use in this study [17]. Consequently, we devised the aforementioned two questions to assess the impact on personal health management and potential influence on others. We deemed the app acceptable if 60% or more of the participants responded positively.

### 2.6. Potential Efficacy

Potential efficacy was gauged by comparing frailty phenotype scores, physical fitness, questionnaire responses, dietary variety scores, and physical activity parameters between pre- and post-intervention phases.

1. Frailty Phenotype Score:

The frailty phenotype score, with one point allocated for each of the following criteria for a total of five points, and the reversal rate of each assessment item were evaluated according to the revised J-CHS criteria [11]. Criteria include shrinking (weight loss of 2–3 kg or more in six months), muscle weakness (grip strength less than 28 kg in men and 18 kg in women), exhaustion (feeling tired for no reason over the past two weeks), slowness (usual walking speed less than 1.0 m/s), and low activity (negative responses to both questions about “Do you engage in moderate levels of physical exercise or sports aimed at health?”, “Do you engage in low levels of physical exercise aimed at health?”).

2. Physical Fitness Test:

Physical fitness was assessed using the chair stand test, the 8-foot-up-and-go test, and the step-in-place test (2 min) from the Senior Fitness Test.

3. Questionnaire Survey:

Health-related quality of life was measured with the 36-item short-form health survey (SF-36) questionnaire [18]. The SF-36 scores are divided into three components: physical, mental, and the role–social component summary (RCS). Additional assessments included the Home-Exercise Barrier Self-Efficacy Scale [19], social isolation via the Japanese version of the Lubben Social Network Scale shortened version, the Geriatric Depression Scale-15 [20], and the dietary variety score (DVS).

4. Step Counts, Physical Activity, and Sedentary Behavior:

Step counts were recorded using the Active style Pro HJA-750C (Omron Healthcare Co., Ltd., Kyoto, Japan), a waist-mounted three-axis accelerometer. Participants were instructed to wear the device on the left lumbar region for one week (excluding times spent bathing, swimming, or sleeping), with a requirement of at least six hours of wear per day. Inclusion in the analysis required a minimum of three days of wear. Measurements of step counts, physical activity, and sedentary behavior were taken at baseline, at the end of week 13, and immediately after the app course (end of week 6).

5. Adverse Events:

A pharmacist serving as a safety evaluation specialist offered an adverse event consultation service. Participants were encouraged to report any incidents or injuries during the study, irrespective of their relation to the application use.

### 2.7. Data Analysis

All statistical analyses were conducted using R (version 4.00). The analysis was two-sided with a significance level set at 0.05 and a 95% confidence coefficient. Continuous data were presented as the number of cases, means, standard deviations, and medians, while frequencies and proportions were tabulated for categorical data. Data from participants who withdrew from the study were excluded. Analyses of potential efficacy were limited to complete data sets, excluding participants from whom data were not obtainable due to equipment failure or refusal to wear the device. For feasibility and acceptability assessments, only participants who completed the post-measures were included.

Furthermore, stratified assessments were conducted for both the intervention (6 weeks) and observation periods (7 weeks). The potential efficacy was determined by calculating changes from baseline and was statistically tested using the *t*-test and Wilcoxon’s rank-sum test. 

## 3. Results

### 3.1. Participants Recruited 

**Figure 2:**
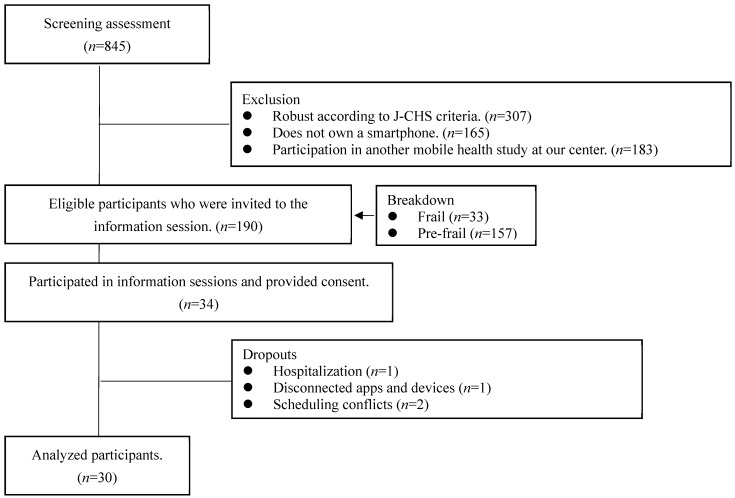
Flow of participant selection.

A total of 845 community-dwelling older adults participated in the screening survey in 2022. Out of these, 190 met the eligibility criteria, after excluding those categorized as robust according to the J-CHS criteria (*n* = 307), non-smartphone users (*n*= 165), and individuals participating in other mHealth studies (*n*= 183). Among those eligible, 34 older adults who attended an on-site research orientation session and provided consent were included in the study (Figure 2).

### 3.2. Baseline Characteristics

Descriptive characteristics of the participants are presented in Table 2. The average age of the participants was 78.15 years (SD = 4.61). Of the thirty-four participants, five were identified as frail. The overall frail phenotype scores were 1.56 (SD = 0.82), with scores of 3.2 (SD = 0.45) for frail participants and 1.28 (SD = 0.45) for pre-frail participants. RCS scores were higher among the pre-frail group. No significant differences were observed in other descriptive characteristics. The participants’ average daily step count was 4898.38 (SD = 2596.04), and the average duration of physical activity per day was 36.90 min (SD = 31.94). 

### 3.3. Attendance and Adverse Events

The study completion rate was 88.2% (30 out of 34 participants completed the course). Reasons for withdrawal included scheduled hospitalizations for surgery unrelated to the study, ownership of smartphone models incompatible with the application, and scheduling conflicts. Participants who withdrew were either frail (n = 1, 20%) or pre-frail (n = 3, 10.3%). The participation rate in the application program among the 30 remaining participants was 100%. No adverse events were reported.

### 3.4. Feasibility and Acceptability

**Figure 3:**
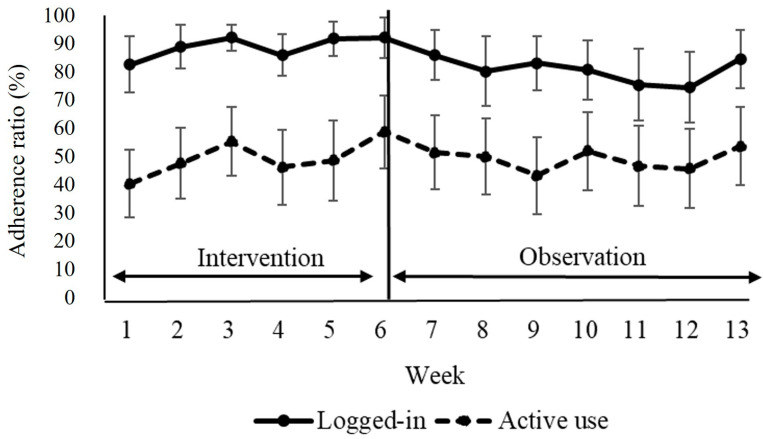
The adherence proportion of app usage, signifying the percentage of participants who logged in at least once per day (logged-in) and the percentage of days when at least seven or more points were acquired (active use) during the 13 weeks.

Figure 3 illustrates the weekly average percentage of logins and active use. The median percentage of logged-in days over the entire period was 88.4% (IQR = 74.5–99.7). During the 6-week intervention period, 95.1% (IQR = 86.0–100) of participants logged in, and during the 7-week observation period, the figure was 95.9% (IQR = 61.1–100). For active use of the app (achieving seven points per day), the percentages were 54.8% (IQR = 24.2–70.5) overall, 54.7% (IQR = 26.8–65.7) during the intervention, and 52.6% (IQR = 25.4–78.6) throughout the observation period. Among the eight participants who fell below the 25th percentile in terms of app logins and active use, two (50% of those identified as frail) were frail and six (23% of those identified as pre-frail) were pre-frail.

Regarding the app’s acceptability, 73% of the participants responded with “agree” or “somewhat agree” to the question, “Does the app help you improve your health?”. Furthermore, 40% of participants responded with “agree” or “somewhat agree” when asked if they would recommend the app to friends and family.

### 3.5. Potential Efficacy

J-CHS scores decreased by 0.40 points (95% confidence interval: −0.73 to −0.07) over the 13 weeks (Table 3). Improvement was also observed in gait speed, with an increase of 0.25 m/s (0.16, 0.34); the 30-s chair stand, with 1.67 more times (0.22, 3.12); and the 8-foot up and go, which decreased by −0.45 s (−0.66, −0.24). However, certain fitness measures deteriorated: grip strength decreased by −1.12 kg (−1.97, −0.27), and the 2-min step test decreased by −4.93 times (−9.03, −0.84).

The SF-36, Home-Exercise Barrier Self-Efficacy Scale, social isolation, Geriatric Depression Scale, and dietary variety score (DVS) did not exhibit significant changes after the intervention.

### 3.6. Step Counts, Physical Activity, and Sedentary Behavior

Step counts did not change after the 6-week intervention period, with a change of 828 steps (−916, 2572). However, an increase of 2069 steps (225, 3913) was observed after the entire observation period (Table 4). As this change was influenced by the time the accelerometer was worn, the value of the change in steps per hour of wear was calculated, and an increase of 132 (33, 231) steps was observed after the end of the period. Nonetheless, no clear changes were noted in time spent in light- (−0.2 (−1.7, 1.2)) or moderate-to-vigorous-intensity physical activity (0.68 (−0.14, 1.5)), or in sedentary time (−0.5 (−2.1, 1.2)) after the intervention period.

## 4. Discussion

This study evaluated the feasibility and acceptability of the mHealth app, “Online Kayoinoba”, among community-dwelling older adults with frailty. Results indicate a login rate of 88.4% throughout the entire period, with over 50% actively using the app. Additionally, 73% of users reported that the app helped them manage their health. However, only 40% of the users agreed to recommend the app to others. Overall, the findings suggest that the “Online Kayoinoba” app is highly feasible, acceptable, and safe for community-dwelling older adults with frailty, particularly for those with pre-frailty, and may positively impact step count maintenance and improvement. Thus, the “Online Kayoinoba” app is a feasible tool that could be utilized in clinical trials to evaluate its effectiveness in promoting self-care and self-management among those with frailty.

Many community-dwelling older adults with frailty used the “Online Kayoinoba” app in our study. The “Online Kayoinoba” application employed in this study was specifically crafted to be user-friendly and intuitive for older adults, enabling them to navigate it effortlessly without any prior instruction (refer to Figure 1). Indeed, it proved to be exceptionally accessible for younger individuals, including the researchers who facilitated the guidance. Although a scoping review by Linn et al. summarized the literature on digital health interventions for older adults with frailty, no interventions using mHealth apps have been documented [10]. The barriers to Internet access and the use of evolving digital devices are significant for older adults [21], especially those with frailty, and further avoidance is expected. However, educating older adults on the use of mHealth applications and Internet literacy has been reported to promote the use of digital devices and generate psychological benefits [22]. Our study did not offer any incentives for participation, yet we recorded a 100% attendance rate. After six lessons, a login rate of over 95% was recorded during the observation period, suggesting the effectiveness of the intervention program and the usability of the mHealth app, “Online Kayoinoba”.

The gamification aspect of “Online Kayoinoba” may contribute to its high feasibility. This study’s application awarded points based on the usage of various functions and daily step counts. Research on gamification-based physical activity promotion is attracting global attention. In addition to increasing physical activity participation, gamification also enhances app engagement [23,24]. This study provides important insights for developing sustainable digital technology intervention strategies to prevent and improve frailty, as no other study has utilized a gamification-powered mHealth application with community-dwelling older adults with frailty. Therefore, although it remains uncertain if gamification significantly motivates the sustained engagement of older adults with frailty in using the mHealth app, the outcomes largely aligned with anticipations based on the app’s specifications and features.

It was also revealed that mHealth apps are perceived as aiding health improvement. Key factors in improving frailty include exercise, a proper diet, and avoiding social isolation. The effectiveness of using mHealth apps for promoting physical activity has become increasingly evident, as observed in this study. mHealth apps, which impose fewer physical constraints, such as not requiring specific exercise locations, may encourage higher engagement than supervised exercise programs in gyms [25]. Additionally, mHealth apps may increase social connectedness, reduce loneliness, and enhance sociability [26]. The “Online Kayoinoba” app, launched nationwide in Japan, includes social networking features, which may influence users’ awareness of their social connections. This contributes to the app’s high feasibility.

The low percentage (40%) of users willing to recommend the app raises concerns about its widespread adoption. Reasons cited in open-ended responses include challenges related to the age and technological proficiency of potential users. The digital divide is a significant social issue, affecting not only different generations but also peers within the same generation. Addressing this requires long-term training to master information and communication technologies [27]. The results underscore the importance of implementing intervention programs alongside mHealth application launches to overcome the digital divide’s barriers. Moreover, the adoption of mHealth apps among vulnerable groups, such as older adults and individuals receiving social psychiatric care, is greatly enhanced by the synergy of close relationships and trust between the target users and the providers, including healthcare professionals and support staff [28]. Consequently, to promote more effective engagement with mHealth apps by older adults, it might be crucial to cultivate avenues for active dialogue with providers or to involve trusted family members of the target demographic as proxy providers.

The use of “Online Kayoinoba” was potentially effective in improving frailty phenotype scores and increasing step counts. The clinical significance of a −0.4-point change in the frailty phenotype remains uncertain, but even slight improvements can potentially decrease the risk of loss of independence due to the dose–response relationship between frailty scores and independence risk [29]. Although participants did not meet the government-recommended step count at baseline, they slightly exceeded it after the intervention [30]. Seasonal variations may influence step counts, suggesting the need for future controlled trials to verify these findings [31].

This study is the first to test the feasibility of an mHealth app for community-dwelling older adults with frailty, providing valuable insights for planning subsequent clinical trials. Improvements in frail phenotype score and physical fitness items suggest potential efficacy, offering foundational information for future trial establishment. However, limitations include the exclusive enrollment of men, the relatively short intervention period, and the lack of a control group. Specifically, the absence of a control group in our study leaves the potential for various biases, including selection bias, performance bias, detection bias, and attrition bias. Consequently, this limitation precludes a thorough exploration of potential efficacy within the confines of this study. To address these concerns, the establishment of a control group and the execution of a randomized trial are required for subsequent research phases. Future studies should consider these factors when planning tests. Additionally, the study’s reliance on self-administered surveys introduces notable limitations concerning reliability and validity, given their dependence on subjective participant responses. Questions such as “Does the app help you improve your health?” can elicit a range of interpretations, suggesting diverse causal mechanisms and potentially compromising the objectivity of the findings due to inconsistent data and inherent biases. Although these surveys provide valuable perspectives, the lack of contextual clarity may diminish the validity of interpretations. Recognizing these limitations highlights the importance of employing mixed-methodology approaches [28] in future research to ensure a more thorough and nuanced analysis, thus enriching the academic discourse surrounding this topic.

## 5. Conclusions

The mHealth app, “Online Kayoinoba”, demonstrated high feasibility for community-dwelling older adults with frailty and potential efficacy for improving frailty phenotype scores and step counts. These findings suggest that tools promoting self-management among independent community-dwelling older adults with frailty may serve as an acceptable foundation for developing sustainable care prevention strategies. Furthermore, clinical trials to test these applications’ effectiveness are well-supported.

## Figures and Tables

**Table 1:** Contents of “Online Kayoinoba” lessons.

Visit	Contents of Lesson	Summary
1	Let us take a walk using the walking function	Installation of the application and explanation of GO points. Start the walking function and try to generate a walking course automatically.
2	1. Let us communicate within the application 2. Review of the walking function	Post, like, and comment on photos using communication tools.
3	1. Enter today’s meal in the diet diary 2. Review of communication tools	Register what you ate this morning (or for lunch) and check your nutritional intake.
4	1. Let us try some local exercises 2. Review the diet check	Participants can access and try more than 1000 different local gymnastics exercises through the app
5	1. Play games using your brain 2. Review of local exercises	Activate and try playing the cognitive game.
6	General review section	Reconfirm understanding of previous functions as a general review of 1–5.

**Table 2:** Descriptive characteristics of participants.

	Overall (*n* = 34)	Pre-Frail (*n* = 29)	Frail (*n* = 5)
Age, year	78.15 (4.61)	77.72 (3.97)	80.60 (7.50)
Body mass index, kg/m^2^	21.75 (3.45)	21.72 (3.61)	21.94 (2.62)
Systolic blood pressure, mmHg	125.29 (16.28)	124.97 (15.74)	127.20 (21.15)
Frail phenotype score	1.56 (0.82)	1.28 (0.45)	3.20 (0.45)
Grip strength, kg	18.01 (3.89)	18.49 (3.62)	15.24 (4.65)
Gait speed, m/s	1.32 (0.20)	1.35 (0.20)	1.18 (0.17)
Chair stand 30 s, times	15.41 (5.73)	15.69 (5.41)	13.80 (7.85)
8-foot up and go, s	6.45 (1.71)	6.25 (1.56)	7.62 (2.23)
2-min step in place, times	112.47 (14.16)	112.45 (14.87)	112.60 (10.33)
Comorbidities, yes			
Hypertension	13 (38.2)	11 (37.9)	2 (40.0)
Stroke	3 (8.8)	2 (6.9)	1 (20.0)
Heart disease	4 (11.8)	4 (13.8)	0 (0.0)
Diabetes	2 (5.9)	2 (6.9)	0 (0.0)
Dyslipidemia	8 (23.5)	7 (24.1)	1 (20.0)
Osteoporosis	9 (26.5)	7 (24.1)	2 (40.0)
Chronic pain, yes			
Low back	5 (14.7)	5 (17.2)	0 (0.0)
Knee	10 (29.4)	9 (31.0)	1 (20.0)
Falls, yes	13 (38.2)	10 (34.5)	3 (60.0)
Home-Exercise Barrier Self-Efficacy Scale	18.15 (3.95)	18.31 (4.10)	17.20 (3.11)
Social isolation, pts	13.88 (4.74)	14.28 (4.80)	11.60 (4.10)
GDS-15	3.21 (2.53)	3.28 (2.70)	2.80 (1.30)
Dietary variety score	4.91 (2.08)	4.90 (1.86)	5.00 (3.39)
SF-36			
PCS	42.70 (9.07)	43.34 (7.99)	38.98 (14.53)
MCS	56.06 (6.57)	55.10 (6.18)	61.66 (6.54)
RCS	48.31 (11.11)	49.79 (10.56)	39.68 (11.35)
Living alone, yes	14 (41.2)	11 (37.9)	3 (60.0)
Wearing time, min/day	947.72 (130.46)	937.83 (106.61)	1003.11 (234.37)
Step counts, steps/day	4898.38 (2596.04)	4906.84 (2666.48)	4851.06 (2432.86)
Light-intensity physical activity, min/day	348.28 (75.99)	341.59 (78.50)	385.74 (50.43)
Moderate-to-vigorous intensity, min/day	36.90 (31.94)	39.35 (33.60)	23.17 (16.36)
Sedentary time, min/day	562.54 (130.24)	556.89 (121.79)	594.20 (184.68)

Data were expressed as mean or median (standard deviation or interquartile ranges). Chronic pain was defined as pain that lasted more than 3 months. Falls were defined as those occurring within one year. GDS-15: Geriatric Depression Scale-15, SF-36: 36-item short-form health survey, PCS: the physical component summary, MCS: the mental component summary, RCS: the role–social component summary.

**Table 3:** Mean changes in parameters (n = 26).

	Baseline to 13 Weeks
Frail phenotype score	−0.40 (−0.73, −0.07)
Grip strength, kg	−1.12 (−1.97, −0.27)
Gait speed, m/s	0.25 (0.16, 0.34)
Chair stand 30 s, times	1.67 (0.22, 3.12)
8-foot up and go, s	−0.45 (−0.66, −0.24)
2-min step in place, times	−4.93 (−9.03, −0.84)
Home-Exercise Barrier Self-Efficacy Scale	−1.63 (−3.41, 0.14)
Social isolation	0.20 (−0.94, 1.34)
GDS-15	−0.23 (−1.14, 0.67)
Dietary variety score	−0.43 (−0.91, 0.04)
SF-36	
PCS	0.89 (−1.51, 3.29)
MCS	0.43 (−1.47, 2.33)
RCS	0.54 (−3.04, 4.12)

Data were expressed as mean change (95% confidence interval). GDS-15: Geriatric Depression Scale-15, SF-36: 36-item short-form health survey, PCS: the physical component summary, MCS: the mental component summary, RCS: the role–social component summary.

**Table 4:** Mean changes in step counts, physical activity, and sedentary behavior (n = 24).

	Baseline to 6 Weeks	Baseline to 13 Weeks
Wearing time, min/day	−14 (−32, 4.2)	−23 (−41, −4.9)
Step counts, steps/day	828 (−916, 2572)	2069 (225, 3913)
Mean change per hour wearing time	41 (−40, 123)	132 (33, 231)
Light-intensity physical activity, min/day	−24 (−38, −10)	−13 (−35, 7.8)
Mean change per hour wearing time	−1.20 (−2.2, −0.24)	−0.2 (−1.7, 1.2)
Moderate-to-vigorous intensity, min/day	8 (−7.1, 23)	11 (−4.2, 27)
Mean change per hour wearing time	0.41 (−0.26, 1.1)	0.68 (−0.14, 1.5)
Sedentary time, min/day	2 (−18, 23)	−21 (−50, 8.8)
Mean change per hour wearing time	0.80 (−0.25, 1.8)	−0.5 (−2.1, 1.2)

Data were expressed as mean change (95% confidence interval).

## Data Availability

The raw data supporting the conclusions of this article will be made available by the authors on request.
